# Editorial: Promising roles of functional RNAs in tuberculosis

**DOI:** 10.3389/fimmu.2023.1164549

**Published:** 2023-03-07

**Authors:** Jiang Pi, Ling Shen, Hongbo Shen

**Affiliations:** ^1^ Guangdong Provincial Key Laboratory of Medical Molecular Diagnostics, The First Dongguan Affiliated Hospital, Guangdong Medical University, Dongguan, China; ^2^ Department of Microbiology and Immunology, University of Illinois at Chicago, Chicago, IL, United States; ^3^ Clinic and Research Center of Tuberculosis, Shanghai Key Lab of Tuberculosis, Shanghai Pulmonary Hospital, Tongji University School of Medicine, Shanghai, China

**Keywords:** functional RNAs, tuberculosis, immunity, vaccine, therapy, diagnosis

Tuberculosis (TB) is induced by *Mycobacterium tuberculosis* (Mtb) infection and has become one of the top killers among infectious diseases. Traditional anti-TB chemotherapy has shown low efficacy in TB control, mainly due to the prevalence of virulent and multidrug-resistant Mtb, which urges us to pay more attention to exploring the precise immune responses involved in Mtb infection so that we can identify novel therapeutic targets. As a nucleic acid, ribose nucleic acid (RNA) plays various essential biological roles that are widely involved in disease development, namely, in gene coding, decoding, regulation, and expression. Recently, different kinds of RNAs, such as messenger RNA (mRNA), long non-coding RNA (lncRNA), and circular RNA (CircRNA), have been found to be associated with TB progression. With the development of new sequencing techniques, more and more functional RNAs have been reported to regulate multiple immune responses during Mtb infection, which positively or negatively regulate anti-TB immunity.

Therefore, new insights into the functions and mechanisms of action for different RNA molecules are of vital importance to finding new targets for TB vaccine, diagnosis, and therapy. Frontiers in Immunology recently published a series of articles under the Research Topic entitled “*Promising Roles of Functional RNAs in Tuberculosis*”. This Research Topic contains two review articles and two original research studies that describe the functions and mechanisms of RNA molecules involved in TB.

Liang et al. review the newest insights into the impact of ncRNAs during Mtb infection. The contribution of ncRNAs to the underlying pathogenesis of Mtb in hosts has been previously evaluated (1). Liang et al., elaborate on the promising roles of ncRNAs in TB and describe the regulatory impact of these ncRNAs on various aspects of immune functions, their impact on Mtb infection, their potential as biomarkers for TB diagnosis, and their impact on Drug-resistant TB (DR-TB) identification and treatment monitoring. Finally, they summarize the available evidence and existing challenges for the use of ncRNAs for TB management, including rapid diagnosis and precise treatment.

Wang et al. introduce recent advances in the immune regulatory roles of circRNAs, as well as their potential diagnostic value in TB. circRNAs are byproducts of aberrant splicing but are now appreciated to have important biological roles, including the regulation of gene expression, the modulation of protein function, and the encoding of proteins (2). In their mini-review, the authors introduce the discovery, biogenesis, and function of circRNAs, and describe the roles of circRNAs in TB and their potential as biomarkers in TB (Wang et al.).

Two original research articles are also included in this Research Topic. Yang et al. screen the differentially expressed genes in TB patients between a healthy cohort and tuberculosis patients by peripheral blood mRNA sequencing. They identify six key genes (AKT1, TP53, EGF, ARF1, CD274, and PRKCZ) and two important miRNAs (has-miR-150-5p and has-miR-25-3p) that could regulate these genes *via* mRNA sequencing. Their results suggest that these six key genes and two important miRNAs may participate in the pathogenesis of infection and invasion of Mtb through herpes simplex virus 1 infection, endocytosis, and B cell receptor signaling pathways.

Sivakumaran et al. assess 35 incipient TB and 12 subclinical TB cases, along with corresponding household active TB cases (n=11) and household controls (n=39) in Southern India, using high throughput methods for transcriptional and protein profiling. They identify an 11-gene signature (ABLIM2, C20orf197, CTC-543D15.3, CTD-2503O16.3, HLADRB3, METRNL, RAB11B-AS1, RP4-614C10.2, RNA5SP345, RSU1P1, and UACA) and an eight protein signature (b-FGF, IFNγ, IL1RA, IL7, IL12p70, IL13, PDGF-BB, and VEGF) that can distinguish subclinical TB from incipient TB with a high discriminatory power by the area under the curve (AUC) in both training and test sets.

In conclusion, these articles provide a systematic overview and deeper understanding of ncRNA, circRNA, and mRNA in the development and immunology of TB, which might benefit the discovery of novel biomarkers in TB. These articles also introduce more evidence that RNA plays critical roles in TB immunity and TB development, although their detailed mechanisms of action need to be investigated further. Clearly, more attention should be paid to the exploration of functional RNAs in TB, especially the exact mechanisms responsible for their functions, which might provide targets for the development of novel anti-TB strategies.

## Author contributions

JP drafted the manuscript. LS and HS revised the manuscript and were responsible for leading this work. All authors contributed to the article and approved the submitted version.
